# Long-term humoral and cellular immunity against vaccine strains and Omicron subvariants (BQ.1.1, BN.1, XBB.1, and EG.5) after bivalent COVID-19 vaccination

**DOI:** 10.3389/fimmu.2024.1385135

**Published:** 2024-05-02

**Authors:** Hakjun Hyun, Eliel Nham, Hye Seong, Jin Gu Yoon, Ji Yun Noh, Hee Jin Cheong, Woo Joo Kim, Sun Kyung Yoon, Se-Jin Park, WonSeok Gwak, June-Woo Lee, Byoungguk Kim, Joon Young Song

**Affiliations:** ^1^ Department of Infectious Diseases, Ajou University School of Medicine, Suwon, Republic of Korea; ^2^ Division of Infectious Diseases, Department of Internal Medicine, Korea University College of Medicine, Seoul, Republic of Korea; ^3^ Asia Pacific Influenza Institute, Korea University College of Medicine, Seoul, Republic of Korea; ^4^ Department of Research and Development, Vaccine Innovation Center, Korea University College of Medicine, Seoul, Republic of Korea; ^5^ Division of Vaccine Clinical Research, Center for Vaccine Research National Institute of Infectious Diseases, Korea National Institute of Health, Korea Disease Control and Prevention Agency, Cheongju, Republic of Korea

**Keywords:** SARS-CoV-2, vaccines, humoral immunity, cellular immunity, durability, EG.5, cross-reactivity

## Abstract

**Background:**

The assessment of long-term humoral and cellular immunity post-vaccination is crucial for establishing an optimal vaccination strategy.

**Methods:**

This prospective cohort study evaluated adults (≥18 years) who received a BA.4/5 bivalent vaccine. We measured the anti-receptor binding domain immunoglobulin G antibody and neutralizing antibodies (NAb) against wild-type and Omicron subvariants (BA.5, BQ.1.1, BN.1, XBB.1 and EG.5) up to 9 months post-vaccination. T-cell immune responses were measured before and 4 weeks after vaccination.

**Results:**

A total of 108 (28 SARS-CoV-2-naïve and 80 previously infected) participants were enrolled. Anti-receptor binding domain immunoglobulin G (U/mL) levels were higher at 9 months post-vaccination than baseline in SAR-CoV-2-naïve individuals (8,339 vs. 1,834, p<0.001). NAb titers against BQ.1.1, BN.1, and XBB.1 were significantly higher at 9 months post-vaccination than baseline in both groups, whereas NAb against EG.5 was negligible at all time points. The T-cell immune response (median spot forming unit/10^6^ cells) was highly cross-reactive at both baseline (wild-type/BA.5/XBB.1.5, 38.3/52.5/45.0 in SARS-CoV-2-naïve individuals; 51.6/54.9/54.9 in SARS-CoV-2-infected individuals) and 4 weeks post-vaccination, with insignificant boosting post-vaccination.

**Conclusion:**

Remarkable cross-reactive neutralization was observed against BQ.1.1, BN.1, and XBB.1 up to 9 months after BA.4/5 bivalent vaccination, but not against EG.5. The T-cell immune response was highly cross-reactive.

## 1 Introduction

Since its first emergence in December 2019, the severe acute respiratory syndrome coronavirus 2 (SARS-CoV-2) has been circulating worldwide, despite global efforts to contain it. As of February 2024, approximately 500,000 cases of coronavirus disease 2019 (COVID-19) and 10,000 deaths from COVID-19 have been reported worldwide each month (1). Since the emergence of the Omicron variant in late 2021, its subvariants have spread widely and persisted (2).

In terms of public health, vaccination is the most effective measure for reducing the morbidity and mortality of COVID-19. The vaccine-induced immunity would be variable depending on the host and environmental factors such as age, sex, comorbidities, pre-existing immune status, vaccine formulation and vaccination interval (3–6). Bivalent COVID-19 vaccines targeting the wild-type (WT) SARS-CoV-2 and Omicron subvariants (BA.4/BA.5 or BA.1) have been developed to enhance immunity against the Omicron subvariants (7). In October 2022, when bivalent COVID-19 vaccines were first introduced in South Korea, the predominant subvariant in South Korea was BA.5, which was gradually replaced by BN.1. BN.1 was the most prevalent subvariant from December 2022 to March 2023 and was replaced by the XBB sublineages thereafter (2).

The real-world effectiveness of bivalent COVID-19 vaccines was found to vary depending on the study period (8–10). A study in Israel from September 2022 to January 2023 found a vaccine effectiveness of 72% against hospitalization (8). However, a study conducted in the UK from December to April 2023 found a lower vaccine effectiveness against hospitalization, ranging from 29.7% to 52.7%, depending on the prevalence of the circulating SARS-CoV-2 variants (9). These studies, which utilized healthcare databases, had a serious disadvantage in that they relied on reported cases. In situations without strict surveillance for SARS-CoV-2 infection, a large number of COVID-19 cases might not be laboratory-confirmed, particularly in settings where there is limited availability of effective antiviral agents. Thus, it is challenging to estimate the effectiveness of vaccines against SARS-CoV-2 infection, which is of paramount importance for the suppression of the ongoing COVID-19 pandemic. Considering the rapid evolution of SARS-CoV-2 and waning immunity over time, it is essential to assess the long-term and cross-reactive immunity against viral variants to establish optimal vaccination strategies.

Neutralizing antibodies (NAb) are known to protect against SARS-CoV-2 infection, whereas cellular immunity might reduce the severity of SARS-CoV-2 infection (11–13). To date, few studies have analyzed the long-term humoral and cellular immunity after receiving the bivalent COVID-19 vaccines, especially after immunization with the new bivalent COVID-19 vaccines. In this study, we aimed to evaluate the long-term humoral immunity up to 9 months post-vaccination as well as cross-reactive humoral/cellular immunity against diverse Omicron subvariants after bivalent COVID-19 vaccination.

## 2 Methods

### 2.1 Study design and procedures

This prospective cohort study was conducted at Korea University Guro Hospital from October 2022 to August 2023. We recruited adults (≥18 years) who had completed the primary series of COVID-19 vaccination and were scheduled to receive a bivalent COVID-19 vaccine (BNT162b2, Pfizer–BioNTech) containing antigens of WT SARS-CoV-2 and BA.4/BA.5 strains. We classified the participants into SARS-CoV-2-naïve (Group 1) and previously SARS-CoV-2-infected (Group 2) individuals. We defined prior SARS-CoV-2-infected participants as those with a known date of COVID-19 diagnosis or a positive anti-nucleocapsid (N) antibody assay result at baseline. Blood samples for immunological analysis were collected longitudinally at baseline (T0), 4 weeks (T1), 3 months (T2), 6 months (T3), and 9 months (T4) after vaccination. Data on demographics (age, sex, and body mass index), comorbidities, and COVID-19-related information (vaccination status and prior SARS-CoV-2 infection) were collected at enrollment.

### 2.2 Immunological analysis

We measured anti-receptor-binding domain (RBD) immunoglobulin G (IgG) antibodies and conducted a focus reduction neutralization test (FRNT) on blood samples to assess the humoral immunity. Anti-RBD IgG antibodies were measured by Elecsys SARS-CoV-2 spike immunoassay (Roche Diagnostics, Basel, Switzerland) using Cobas 8000 (Roche, Basel, Switzerland) according to the manufacturer’s protocol (14). A FRNT was performed using WT (bCoV/Korea/KCDC03/2020 NCCP No. 43326), BA.5 (GRA: BA.5 NCCP No. 43426), BQ.1.1 (GRA: BQ.1.1 NCCP No. 43427), BN.1 (GRA: BN.1 NCCP No. 43439), XBB.1 (GRA: XBB.1 NCCP No. 43428), and EG.5 (GRA: EG.5 NCCP No.43455) variants of SARS-CoV-2. Anti-RBD IgG antibodies were measured at T0, T1, T2, T3, and T4 in all participants. Initially, neutralization assays were conducted against the vaccine strains (WT and BA.5 strains) at T0, T1, and T3 and Omicron subvariants (BQ.1.1, BN.1, XBB.1) at T0 and T1 in all participants. After the emergence of EG.5, additional neutralization assay was conducted for the randomly selected 30 participants to compare cross-reactive immunity against WT, BA.5, BQ.1.1, BN.1, XBB.1, and EG.5 at each time point (T0, T1, T3 and T4) as presented in **Supplementary Table 1**. Using the SARS-CoV-2 IgG assay (Abbott Laboratories, Abbott Park, IL, USA), an anti-N antibody assay was conducted on all participants at T0 and for those who had negative results at the previous visit at each time point thereafter. An interferon-gamma enzyme-linked ImmunoSpot (ELISpot) assay was used to evaluate the cross-reactive T-cell immune responses against WT, BA.5, and XBB.1.5 at T0 and T1 in randomly selected 25 participants. Details of the immunological analyses (FRNT and ELISpot assay) are presented in the **Supplementary Materials**. Breakthrough infections in the participants were identified by self-reports and seroconversion of anti-N antibodies. Data obtained after breakthrough infections were excluded from analysis.

### 2.3 Statistical analysis

Geometric mean titers (GMTs) with 95% confidence intervals (CIs) of the anti-RBD IgG antibodies and neutralizing antibodies were calculated after logarithmic transformation of the antibody titers. The INF-γ ELISpot responses were reported as medians with interquartile ranges (IQR). The Wilcoxon signed-rank test was used to compare two paired groups, and the Mann–Whitney U test was used to compare two unpaired groups. Friedman and Kruskal–Wallis tests were used to compare three paired and unpaired groups, respectively. Statistical analyses were performed using SPSS version 20 (IBM Corp., Armonk, NY, USA) or GraphPad Prism version 5.0 (GraphPad Software, Inc., San Diego, CA, USA). Statistical significance was set at p < 0.05.

### 2.4 Sample size

The sample size was calculated using G*power 3.1.9.7. software with an effect size of 0.8, two-tailed alpha error probability of 0.05, and power level of 0.95 (15). The minimum sample size for Wilcoxon signed-rank test was determined to be 21. For Mann–Whitney U test with allocation ratio of 3, the minimum sample size was determined to be 26 for Group 1 and 76 for Group 2.

### 2.5 Ethics statement

This study was approved by the Ethics Committee of Korea University Guro Hospital (2021GR0099) and was conducted in accordance with the Declaration of Helsinki and Good Clinical Practice guidelines. Written informed consent was obtained from all the participants.

## 3 Results

### 3.1 Study participants

A total of 111 participants were recruited and after excluding 3 participants who withdrew their consent, 108 participants (mean age, 46.3 years) were included in the analysis. Based on prior SARS-CoV-2 infection, 28 and 80 participants were classified into Groups 1 and 2, respectively. Sex, age, and comorbidities were statistically indistinguishable between the two groups (**Table 1**). Overall, 66.7% of the participants had received a primary series of COVID-19 vaccination with an adenovirus-vector vaccine, and this proportion was higher in Group 2 than in Group 1 (72.5% vs. 50.0%, p = 0.030). In addition, most participants (93.5%) had received a single booster dose of a monovalent mRNA vaccine before administration of the bivalent mRNA vaccine. The mean interval between the last dose of WT monovalent COVID-19 vaccine to bivalent vaccination was 351 ± 59 days and did not differ between the two groups. Of the 66 participants in Group 2 who knew the date of their COVID-19 diagnosis, the mean interval between prior SARS-CoV-2 infection and bivalent vaccination was 239 ± 66 days. All participants completed the 9-month follow-up. During follow-up, 84 participants were not infected with SARS-CoV-2, and a total of 24 cases of breakthrough infection (14 cases from Group 1 and 10 cases from Group 2) were identified: 6 cases between T0 and T1, 7 cases between T1 and T2, 6 cases between T2 and T3, and 5 cases between T3 and T4.

**Table 1:** Baseline characteristics of the study participants.

	Group 1 SARS-CoV-2-naïve (n=28)	Group 2 Prior SARS-CoV-2-infected (n=80)	*P*-value
Demographics			
Male, n (%)	11 (39.3)	25 (31.3)	0.438
Mean age ± 95% CI, y	46.7 ± 11.7	46.1 ± 10.5	0.808
Mean BMI ± 95% CI, kg/m^2^	23.7 ± 3.7	22.8 ± 3.4	0.225
Comorbidities, n (%)			
Chronic heart disease	0 (0)	1 (1.3)>	> 0.999
Chronic lung disease	0 (0)	1 (1.3)	> 0.999
Chronic liver disease	0 (0)	1 (1.3)	> 0.999
Chronic renal disease	0 (0)	0 (0)	> 0.999
Rheumatologic disease	0 (0)	3 (3.8)	0.567
Hypertension	4 (14.3)	10 (12.5)	0.754
Diabetes mellitus	1 (3.6)	6 (7.5)	0.674
Dyslipidemia	5 (17.9)	6 (7.5)	0.148
Priming COVID-19 vaccine, n (%)			0.030
Adenovirus-vector vaccine^a^	14 (50.0)	58 (72.5)	
ChAdOx1 nCOV-19	14 (50.0)	56 (70.0)	
Ad26.COV2.S	0 (0)	2 (2.5)	
mRNA vaccine^b^	14 (50.0)	22 (27.5)	
BNT162b2	2 (7.1)	4 (5.0)	
mRNA-1273	12 (42.9)	18 (22.5)	
Number of prior vaccine doses, n (%)			0.706
0	1 (3.6)	1 (1.3)	
1	26 (92.9)^c^	75 (93.8)^d^	
2	1 (3.6)^e^	4 (5.0)^f^	
Mean interval between previous dose and bivalent vaccination, days ± 95% CI	349 ± 63	351 ± 59	0.870

a ChAdOx1 nCOV-19 (AstraZeneca) or Ad26.COV2.S (Janssen).

b BNT162b2 (Pfizer–BioNTech) or mRNA-1273 (Moderna).

c BNT162b2 (n=23), mRNA-1273 (n=3).

d BNT162b2 (n=70), mRNA-1273 (n=5).

e BNT162b2–BNT162b2 (n=1).

f BNT162b2–BNT162b2 (n=2), BNT162b2–mRNA-1273 (n=1), mRNA-1273–mRNA-1273 (n=1).

BMI, body mass index; CI, confidence interval.

### 3.2 Humoral immune response

**Figure 1:**
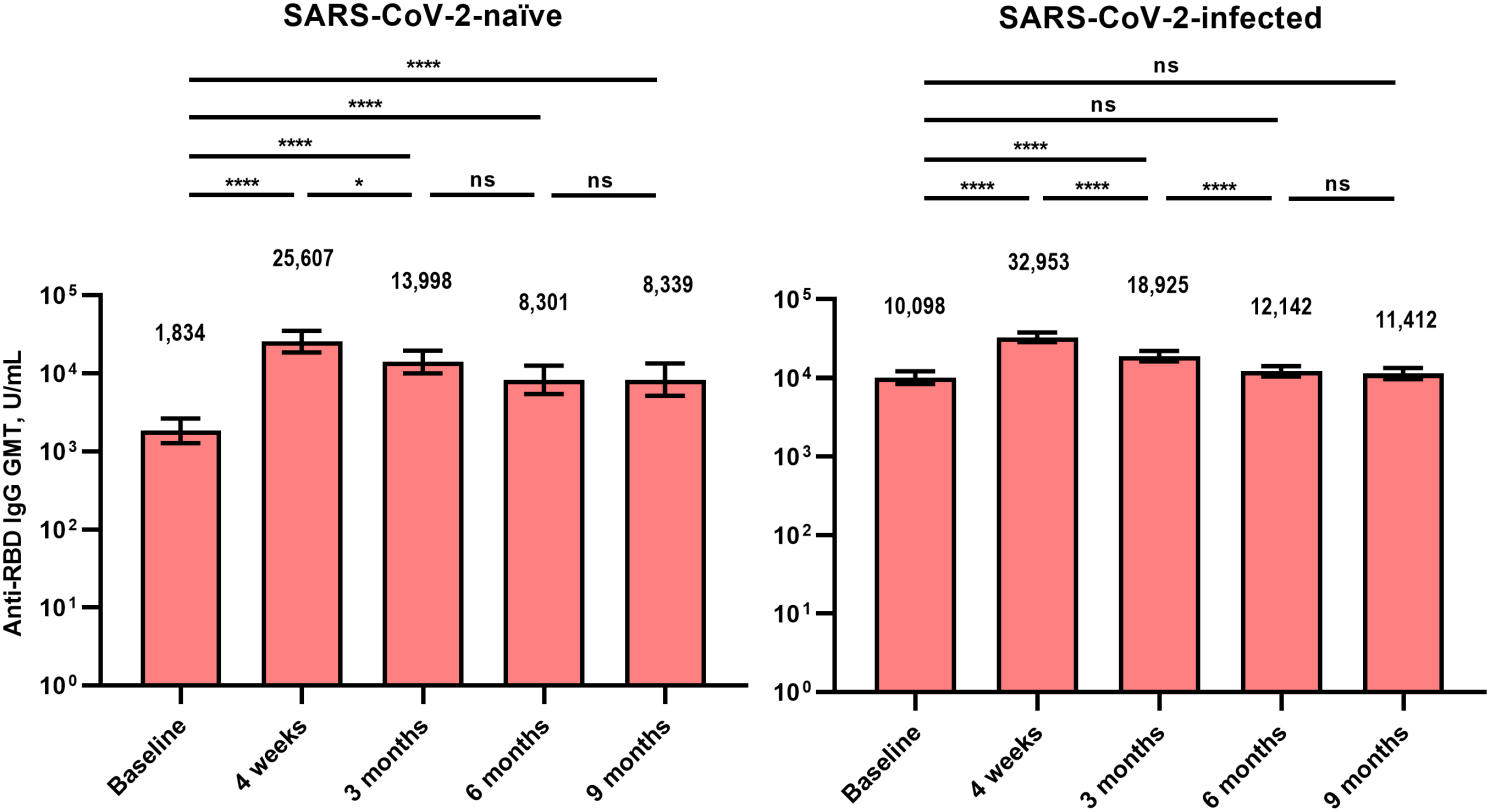
Anti-receptor binding domain immunoglobulin G antibodies after BA.4/5 bivalent mRNA COVID-19 vaccination. The columns represent the geometric mean titers and black bars represent 95% confidence intervals. Statistically significant p-values are marked with asterisks (*p < 0.05, ****p < 0.0001). GMT, geometric mean titer; IgG, immunoglobulin G; ns, not significant; RBD, receptor binding domain.

The GMT of the anti-RBD IgG antibody (U/mL) at T0 was lower in Group 1 than in Group 2 (1,834 vs. 10,098, p < 0.001) (**Figure 1**). In both groups, the anti-RBD IgG antibody titers peaked at T1 and did not differ significantly between Groups 1 and 2 (25,607 vs. 32,953, p = 0.209). The anti-RBD IgG antibody titer remained higher compared with the baseline for up to 9 months after vaccination in Groups 1 (8,339 vs. 1,834, p < 0.001) and 2 (11,412 vs. 10,098, p = 0.708). The GMT of the anti-RBD IgG antibody at T4 did not differ significantly between Groups 1 and 2 (8,339 vs. 11,412, p = 0.083).

**Figure 2:**
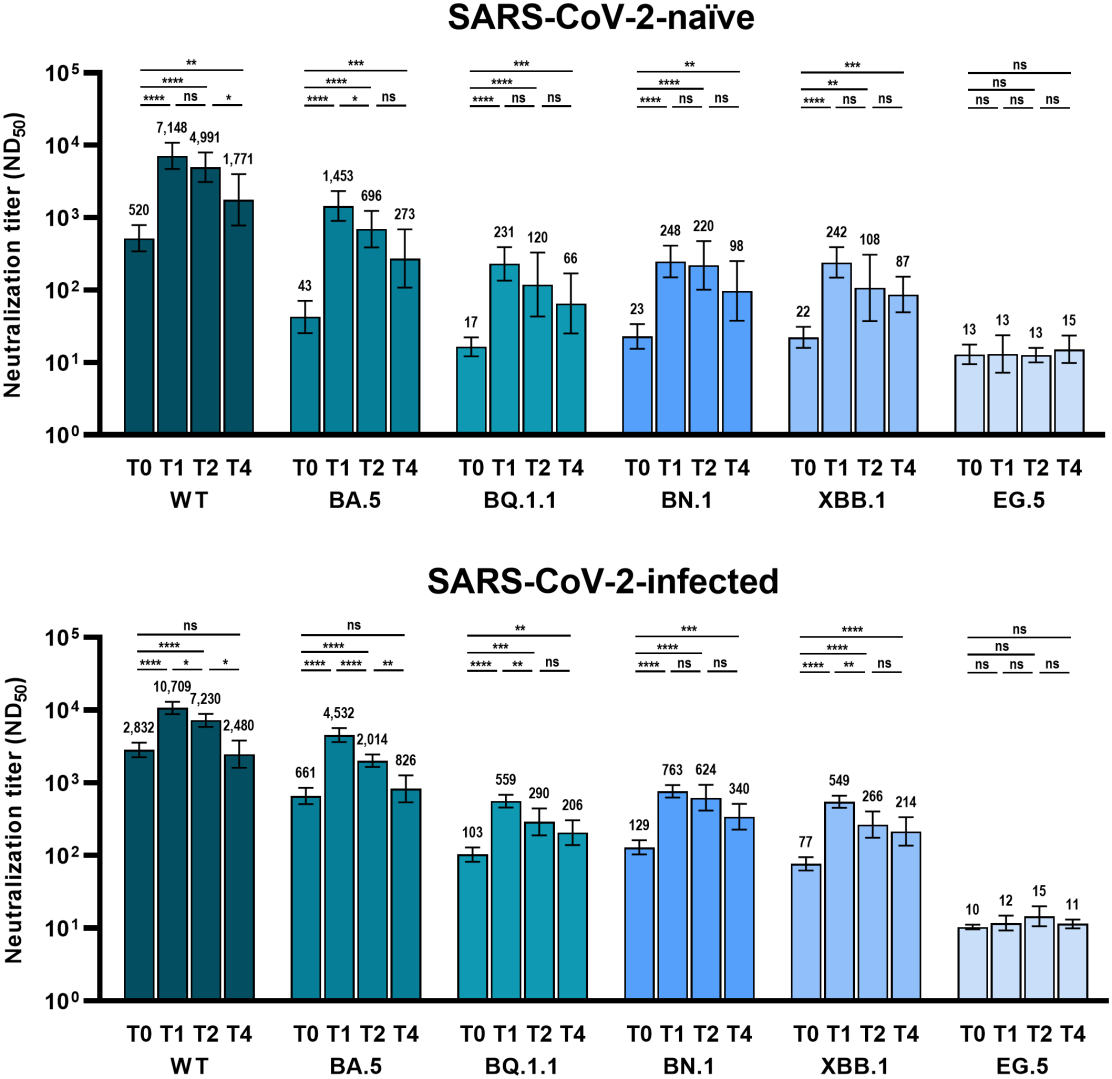
Comparison of neutralizing activity against wild-type and Omicron subvariants at different time points after BA.4/5 bivalent mRNA COVID-19 vaccination. The columns represent the geometric mean titers and black bars represent 95% confidence intervals. Time points are presented as T0 (baseline), T1 (4 weeks after vaccination), T2 (3 months after vaccination), and T4 (9 months after vaccination). Statistically significant p-values are marked with asterisks (*p < 0.05, **p < 0.01, ***p < 0.001, ****p < 0.0001). ND_50_, 50% neutralization dilution; ns, not significant; WT, wild-type.

**Figure 3:**
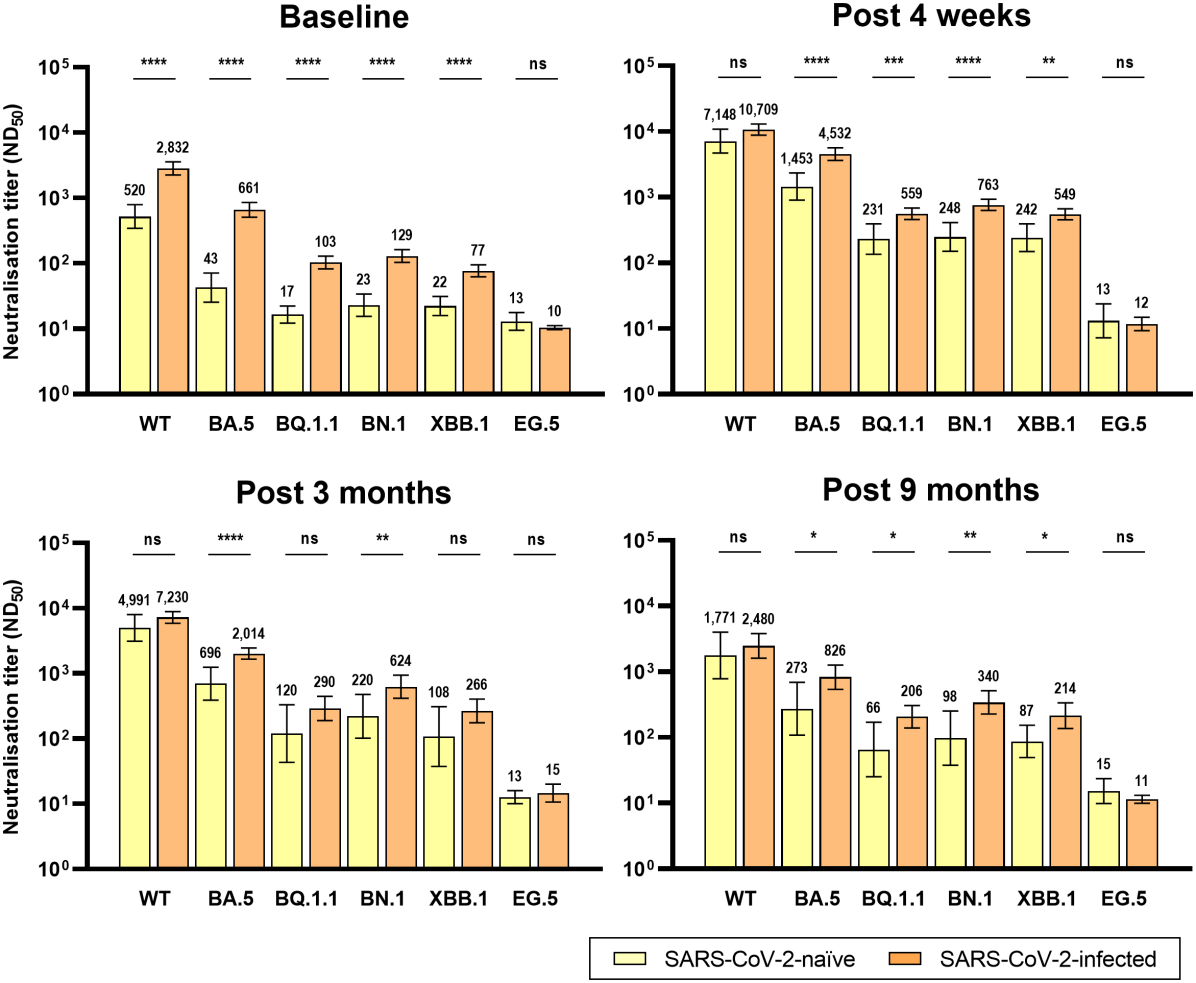
Comparison of neutralizing activity against wild-type and Omicron subvariants by prior SARS-CoV-2 infection after BA.4/5 bivalent mRNA COVID-19 vaccination. The columns represent the geometric mean titers and black bars represent 95% confidence intervals. Statistically significant p-values are marked with asterisks (*p < 0.05, **p < 0.01, ***p < 0.001, ****p < 0.0001). ND_50_, 50% neutralization dilution; ns, not significant; WT, wild-type.

The analyzed number of individuals (neutralization assay) at each time point is presented in **Supplementary Table 2**. Similar to the anti-RBD IgG antibodies, the GMT of NAb after bivalent vaccination peaked at T0 and gradually waned till T4 (**Figure 2**). At T0, the GMTs of NAb against WT, BA.5, BQ.1.1, BN.,1 and XBB.1 SARS-CoV-2 were significantly higher in Group 2 than in Group 1 (**Figure 3**). Neutralizing activity against the Omicron subvariants at T0 was negligibly low in Group 1. In Group 2, cross-reactive neutralizing activity against Omicron subvariants was observed at T0, but the GMT of the NAb against XBB.1 was significantly lower than that of the NAb against BN.1 and BQ.1.1 at T0 (BN.1 vs. BQ.1.1, 103 vs. 129, p = 0.066; BN.1 vs. XBB.1, 129 vs. 77, p < 0.001; XBB.1 vs. BQ.1.1, 77 vs. 103, p = 0.004) (**Supplementary Figure 1**). At T1, after bivalent mRNA vaccination, the NAb titers against WT/BA.5/BQ.1.1/BN.1/XBB.1 in Groups 1 and 2 increased by 13.7/33.8/13.6/10.8/11.0-fold and 3.8/6.9/5.4/5.9/7.1-fold, respectively. The GMTs of NAb against BA.5, BQ.1.1, BN.1, and XBB.1 were significantly higher in Group 2 than in Group 1 at T1 and T4 (**Figure 3**). The neutralizing activity against EG.5 was negligible at T0 and did not increase after bivalent mRNA vaccination in either group.

The GMTs of NAb against BQ.1.1, BN.1, and XBB.1, were significantly higher at T4 than at T0 in both groups. However, the NAb titers against the WT and BA.5 SARS-CoV-2 were significantly higher at T4 than at T0 only in Group 1 (**Figure 2**).

### 3.3 Cellular immune response

**Figure 4:**
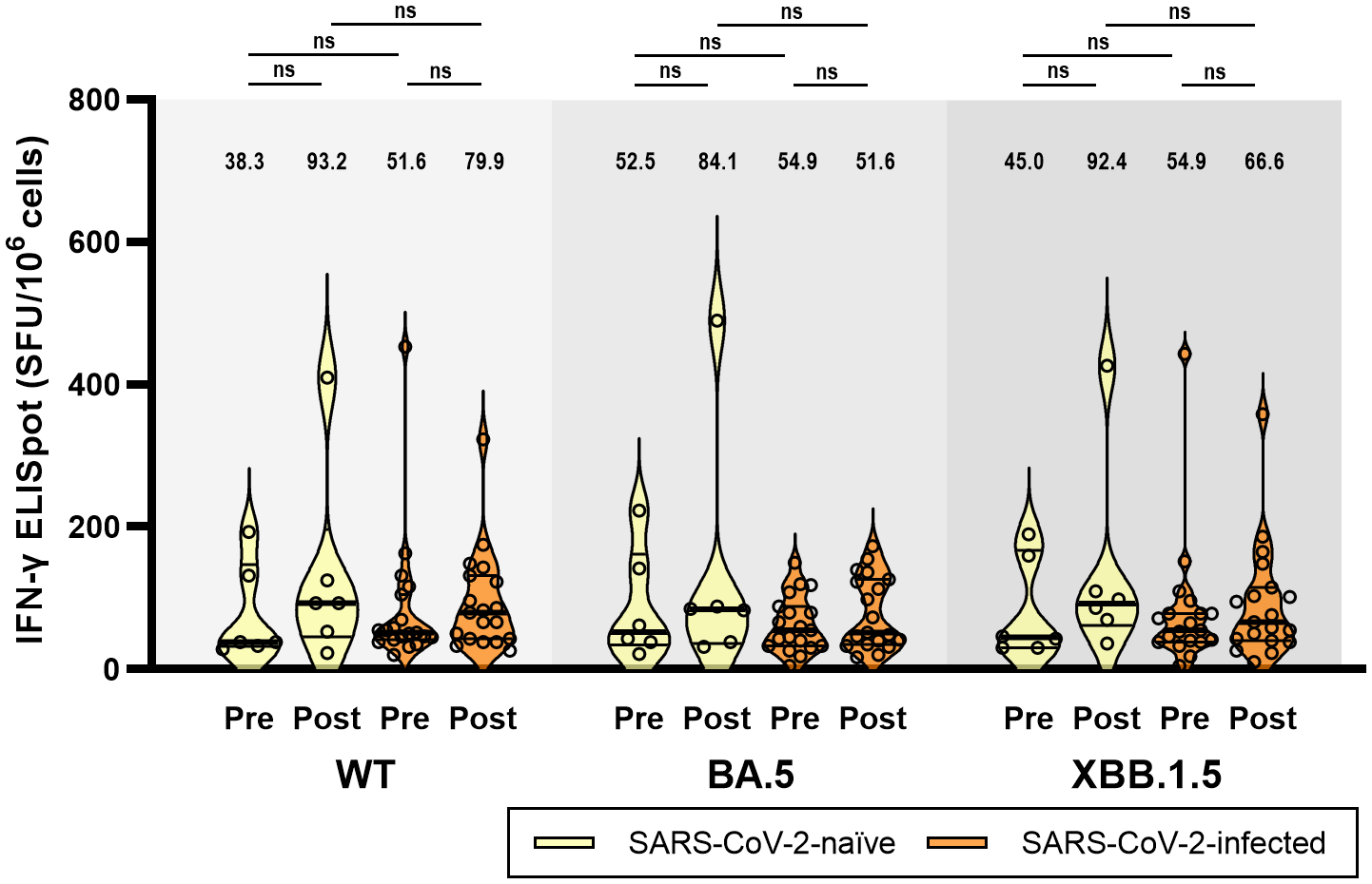
T-cell immune responses against wild-type, BA.5, and XBB.1.5 subvariants of SARS-CoV-2 at baseline (pre-vaccination) and 4 weeks (post-vaccination) after BA.4/5 bivalent mRNA COVID-19 vaccination. The black bar represents the median with interquartile range. ELISpot, enzyme-linked ImmunoSpot; IFN-γ, interferon-γ; ns, not significant; SFU, spot forming unit; WT, wild-type.

An ELISpot assay was conducted to evaluate the T-cell immune responses in six participants from Group 1 and 19 participants from Group 2. In contrast to humoral immunity, the T-cell immune response before vaccination was cross-reactive to Omicron subvariants and did not differ between the two groups. At T0, ELISpot responses (median spot forming unit/10^6^ cells) against WT, BA.5, and XBB.1.5 did not differ in Group 1 (38.3, 52.5, and 45.0, respectively, p = 0.513) and Group 2 (51.6, 54.9, and 54.9, respectively, p = 0.230) (**Figure 4**). Although not statistically significant, the T-cell immune response was enhanced after bivalent mRNA vaccination. However, similar to T0 before vaccination, there was no difference in ELISpot responses against WT, BA.5, and XBB.1.5 at T1 in either Group 1 (93.2, 84.1, and 92.4, respectively, p = 0.511) or Group 2 (79.9, 51.6, and 66.6, respectively, p = 0.223).

## 4 Discussion

This prospective cohort study investigated the cross-reactive and long-term immune responses (up to 9 months) after vaccination with the BA.4/5 bivalent mRNA COVID-19 vaccine. The key findings of this study are as follows: (1) Humoral immunity induced by bivalent mRNA COVID-19 vaccination persists for up to 9 months; (2) Bivalent mRNA COVID-19 vaccine enhanced cross-neutralizing activity against the Omicron subvariants (BA.5, BQ.1.1, BN.1, and XBB.1) and this activity was higher in participants with prior SARS-CoV-2 infection; (3) BA.4/5 bivalent mRNA COVID-19 vaccine did not induce neutralizing activity against the EG.5 variant; (4) and the T-cell immune response was highly cross-reactive against WT and Omicron subvariants at baseline, but T-cell immune boosting was limited with repeated COVID-19 vaccination or SARS-CoV-2 infection.

Vaccine-elicited immunity against SARS-CoV-2 has been challenged by reduced cross-neutralizing activity against new variants, which increases over time (16–19). Accumulating evidence suggests that humoral immunity after vaccination against COVID-19 persists for at least 6 months, whereas the durability of cellular immunity is longer (16–21). A study in a large cohort of 2,964 participants from Japan demonstrated that the rate of antibody waning was reduced with the accumulation of additional doses (22). Therefore, a long-term immunological analysis of novel COVID-19 vaccines is necessary to establish future vaccination strategies. In this study, we found that humoral immunity following bivalent mRNA vaccination persisted for over 9 months, with significantly higher levels than those at baseline. Neutralizing activity against BQ.1.1, BN.1, and XBB.1 was enhanced regardless of prior SARS-CoV-2 infection for up to 9 months. Given that the period between the introduction of bivalent COVID-19 vaccines (October 2022) and dominance of XBB sublineages (August 2023) spans approximately 10 months, this finding suggests the potential benefit for vaccine recipients by enhancing cross-neutralizing activity against emerging variants (2). Data on the durability of immunity following bivalent COVID-19 vaccination are scarce. A recent study by Favresse et al. (23) reported that NAb against BA.5, measured using a pseudovirus neutralization assay, declined 6 months after bivalent vaccination, compared with the baseline. The study by Favresse et al, was conducted primarily in SARS-CoV-2-infected individuals, similar to this study. Notably, in this study, NAb against BA.5 were sustained at higher levels at 9 months post-vaccination compared with those before bivalent COVID-19 vaccination only among SARS-CoV-2-naïve individuals. Another difference between the two studies is the type of NAb assay used. We conducted a live virus-based FRNT. In addition, we found a significant enhancement in the neutralizing activity against diverse Omicron subvariants for up to 9 months.

In this study, the cross-reactive neutralizing activities against Omicron subvariants were higher in the previously SARS-CoV-2-infected participants than SARS-CoV-2-naïve participants. The synergy of natural immunity and vaccine-elicited immunity has been called hybrid immunity, which is known to be better immunogenic (24). Compared to the vaccination alone, hybrid immunity induces a better affinity maturation of memory B cells, producing antibodies with higher potency and broader cross-reactivity (25, 26).

In this study, the neutralizing activity against EG.5 was not enhanced even after administration of the bivalent mRNA vaccine, as previously reported (27–29). EG.5, a descendant of XBB.1.9.2, has an additional mutation, *F456L*, in the spike protein and was designated as a variant of interest in August 2023 (30). XBB variant was derived from BA.2 and had additional five mutations in the N-terminal domain (*V83A*, *del 144*, *H146Q*, *Q183E*, and *V213E*) and five mutations on RBD (*G339H*, *R346T*, *L368I*, *F486S*, and *F490S*). The *F486P* substitution of EG.5, XBB.1.9.2 and XBB.1.5 rather than the *F486S* substitution in other XBB variants is known to enhance transmissibility (31). The mutations in the SARS-CoV-2 RNA genome can induce alteration of epitope which recognized by vaccine or infection-elicited immunity, and they also have the potential to affect the efficacy of therapeutic agents and the sensitivity of molecular tests (32). As XBB.1.9.2 and XBB.1.5 have the same amino acid profiles, the recently updated XBB.1.5 monovalent vaccine could induce strong neutralizing activity against the currently prevalent EG.5 variant and its sublineages (33).

In this study, we observed cross-reactive T-cell immune responses against WT, BA.5, and XBB.1.5, before and after bivalent mRNA vaccination. The positive ELISpot response at baseline may be due to the relatively long half-life of cellular immunity. The half-life of SARS-CoV-2-specific T-cell immunity is longer (approximately 190 days) than that of NAb (approximately 90 days) (21). The lack of remarkable differences in the ELISpot response between SARS-CoV-2-naïve and previously infected individuals might reflect the ceiling effect of repeated vaccination. However, this may also be attributed to the long interval between bivalent vaccine administration and past infection (mean, 249 days) or vaccination (mean, 349 days). The cross-reactivity of the T-cell immune response supports the findings of previous studies, indicating that T-cell epitopes are conserved across various SARS-CoV-2 variants and extend beyond the RBD (17, 34).

This study has several limitations. First, we did not conduct a neutralization assay against Omicron subvariants or an ELISpot assay in all vaccinated individuals. The small sample size may make it difficult to find a significant difference between groups. In particular, we conducted the ELISpot assay on only six participants in Group 1, and a larger sample size might have a chance to reveal significant differences. Therefore, it is necessary to interpret these results carefully and verify their consistency with the findings from other studies. Second, no information was available on the SARS-CoV-2 strains in previously infected individuals. Immune responses vary depending on the prior-infected SARS-CoV-2 strain. Third, the type of priming COVID-19 vaccine received by participants significantly differ between the groups, which could act as a confounding factor. Fourth, we could not exclude the possibility of asymptomatic re-infection in the study participants. Because the rising extent of anti-N IgG antibody titers is not established to detect asymptomatic reinfection, we conducted follow-up anti-N antibody assay only in the SARS-CoV-2 infection-naïve participants. A strength of our study is that we conducted neutralization assays against a series of SARS-CoV-2 variants that emerged for up to 9 months after the introduction of the bivalent vaccine.

In conclusion, bivalent mRNA COVID-19 vaccination induces humoral immunity that persists for up to 9 months. Furthermore, remarkable cross-reactive neutralizing activity was observed against BQ.1.1, BN.1, and XBB.1, but not against EG.5. T-cell immune responses were highly cross-reactive against the WT and diverse Omicron subvariants.

## Data availability statement

The original contributions presented in the study are included in the article/**Supplementary Material**. Further inquiries can be directed to the corresponding author.

## Ethics statement

The studies involving humans were approved by Ethics Committee of Korea University Guro Hospital. The studies were conducted in accordance with the local legislation and institutional requirements. The participants provided their written informed consent to participate in this study.

## Author contributions

HH: Conceptualization, Data curation, Formal Analysis, Investigation, Methodology, Validation, Visualization, Writing – original draft, Writing – review & editing. EN: Investigation, Writing – review & editing. HS: Investigation, Writing – review & editing. JY: Investigation, Writing – review & editing. JN: Investigation, Writing – review & editing. HC: Investigation, Writing – review & editing. WK: Investigation, Writing – review & editing. SY: Investigation, Writing – review & editing, Formal Analysis. SP: Formal Analysis, Investigation, Writing – review & editing. WG: Formal Analysis, Investigation, Writing – review & editing. JL: Formal Analysis, Investigation, Writing – review & editing. BK: Formal Analysis, Investigation, Writing – review & editing, Methodology, Validation. JS: Formal Analysis, Investigation, Methodology, Validation, Writing – review & editing, Conceptualization, Data curation, Funding acquisition, Project administration, Visualization, Writing – original draft.
