# Supplementary material for: Long-term humoral and cellular immunity against vaccine strains and Omicron subvariants (BQ.1.1, BN.1, XBB.1, and EG.5) after bivalent COVID-19 vaccination
Source: Front Immunol. 2024 May 2;15:1385135. doi: 10.3389/fimmu.2024.1385135 (PMC11096540; doi:10.3389/fimmu.2024.1385135)
Supplement: Supplementary file 1 [file DataSheet_1.docx]

Supplementary Material

# Supplementary Data

***Focus reduction neutralization assay***

We diluted serum samples serially from 1:20 to 1:43,740. The mixtures of serum dilution/virus were added into 96-well plates seeded with Vero E6 cells and cultured for 7.5 hour. Immunostaining foci were visualized by sequentially incubating the mixture using SARS-CoV-2 nucleoprotein rabbit monoclonal antibody (Sino Biological, Beijing, China; 1:3000) and a secondary goat anti-rabbit immunoglobulin G horseradish peroxidase-conjugated antibody (Bio-rad, Hercules, CA, USA; 1:2000). The Vero E6 cells were ﬁxed with 4% paraformaldehyde and stained to visualize by TrueBlue Peroxidase substrate (Seracare, Milford, MA, USA). We calculated a reduction in foci count of 50% (FRNT50) using a four-parameter logistic curve ﬁt of SoftMax Pro GxP Software (Version 7.1.2.). The four-parameter logistic equation in the program is as follows: FRNT 50% = InterpX(Plot#1@Graph#1, 50).

***Interferon-γ enzyme-linked ImmunoSpot (ELISpot) assay***

ELISpot plates (Human IFN-γ ELISpotPRO kit, MABTECH, Sweden) were blocked by RPMI medium 1640 (Gibco, NY, USA) containing 10% fetal bovine serum (FBS, Gibco) and 1% penicillin/streptomycin (p/s, Gibco). After washing, the plates were added with 2 μg/well of PepMix™ SARS-CoV-2 spike peptide pools (wild-type, BA.5 or XBB.1.5, JPT Peptide Technologies, Berlin, Germany) and 3 × 10^5^ cells/well of peripheral blood mononuclear cells. Stimulation with DMSO or PMA/Ionomycin was as negative and positive controls, respectively. The plates were then processed following the manufacturer’s protocol and median spot forming units (SFUs) were counted on the ELISpot reader. The results are presented as SFU per million input PBMC (SFU/10^6^ PBMC).

# Supplementary Figures and Tables

## Supplementary Tables

**Supplementary Table 1.** Number of individuals tested for neutralization assay at each time point

| SARS-CoV-2 strain | Baseline, n | 4 weeks post-vaccination, n | 3 months post-vaccination, n | 9 months post-vaccination, n |
| --- | --- | --- | --- | --- |
| Wild-type | 108 | 108 | 108 | 30 |
| BA.5 | 108 | 108 | 108 | 30 |
| BQ.1.1 | 108 | 108 | 30 | 30 |
| BN.1 | 108 | 108 | 30 | 30 |
| XBB.1 | 108 | 108 | 30 | 30 |
| EG.5 | 30 | 30 | 30 | 30 |

**Supplementary Table 2.** Number of individuals included in analysis at each time point in neutralization assay

| SARS-CoV-2 strain | Baseline, n | | 4 weeks post-vaccination, n | | 3 months post-vaccination, n | | 9 months post-vaccination, n | |
| --- | --- | --- | --- | --- | --- | --- | --- | --- |
|  | Group 1 | Group 2 | Group 1 | Group 2 | Group 1 | Group 2 | Group 1 | Group 2 |
| Wild-type | 28 | 80 | 24 | 78 | 21 | 75 | 8 | 15 |
| BA.5 | 28 | 80 | 24 | 78 | 21 | 75 | 8 | 15 |
| BQ.1.1 | 28 | 80 | 24 | 78 | 10 | 18 | 8 | 15 |
| BN.1 | 28 | 80 | 24 | 78 | 10 | 18 | 8 | 15 |
| XBB.1 | 28 | 80 | 24 | 78 | 10 | 18 | 8 | 15 |
| EG.5 | 12 | 18 | 12 | 18 | 10 | 18 | 8 | 15 |

## Supplementary Figures


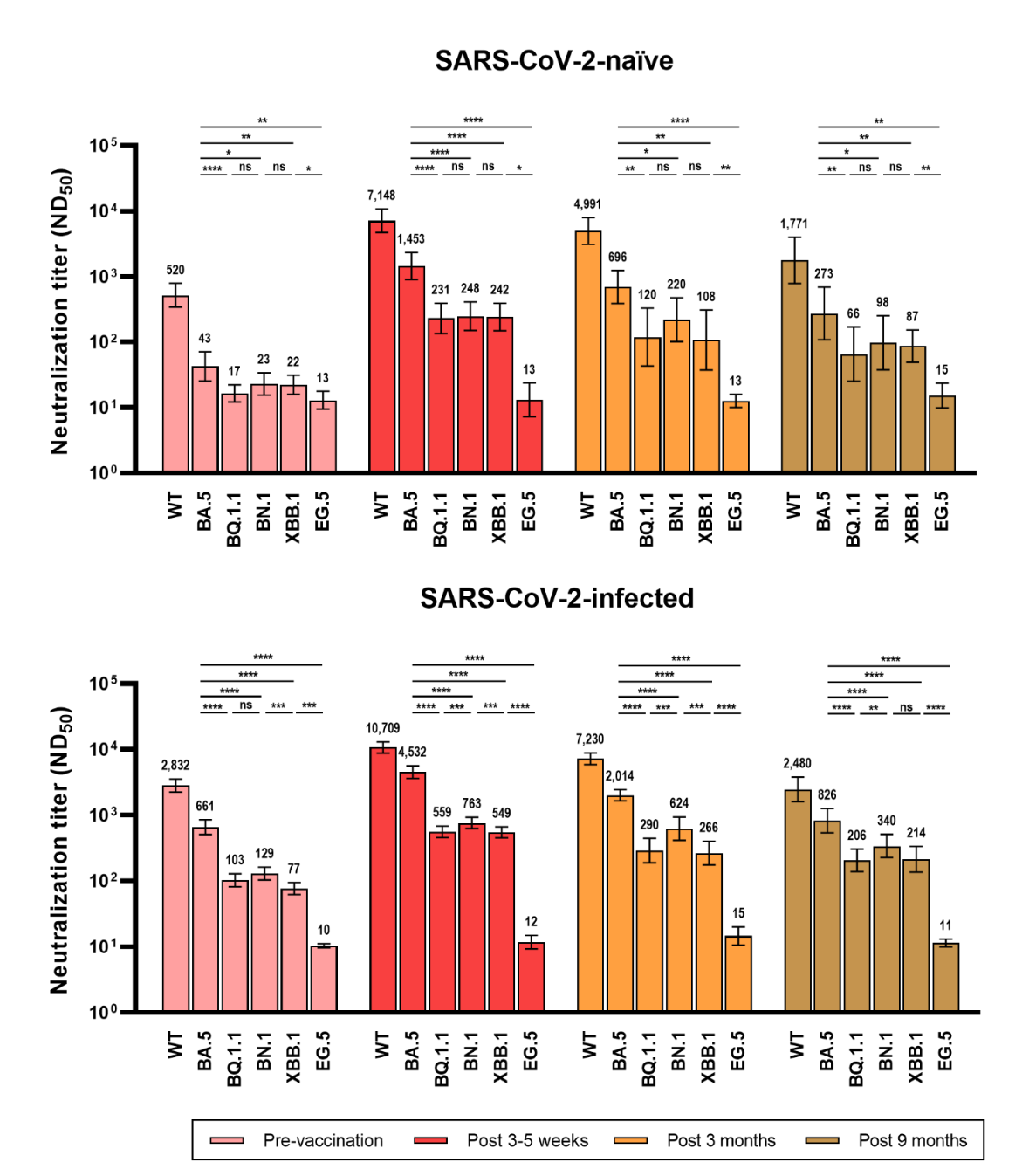


**Supplementary Figure 1.** Comparison of neutralizing activity among wild-type and Omicron subvariants at different time points after the administration of BA.4/5 bivalent mRNA COVID-19 vaccine. The columns represent the geometric mean titers and black bars represent 95% confidence intervals. Statistically significant P-values are marked with asterisks (*p < 0.05, **p < 0.01, ***p < 0.001, ****p<0.0001). Abbreviations: ND_50_, 50% neutralization dilution; WT, wild-type; ns, not significant.
